# Psychological effects of psychedelics in adolescents

**DOI:** 10.3389/frcha.2024.1364617

**Published:** 2024-06-07

**Authors:** Nadhrah Izmi, Robin Lester Carhart-Harris, Hannes Kettner

**Affiliations:** ^1^ Centre for Psychedelic Research, Department of Brain Sciences, Faculty of Medicine, Imperial College London, London, United Kingdom; ^2^ Psychedelics Division, Neuroscape, University of California, San Francisco, San Francisco, CA, United States

**Keywords:** adolescence, adulthood, mental well-being, psychedelics, naturalistic setting, early intervention, mental health

## Abstract

This study aimed to investigate differences in long-term psychological effects, acute subjective effects, and side effects associated with psychedelic use in adolescents (aged 16–24), compared with adults (aged 25+). Data from two observational online survey cohorts was pooled, involving adolescents (average age 20.4 ± 2.2, *N* = 435) and adults (average age 36.5 ± 9.7, range = 25–71, *N* = 654) who self-initiated a psychedelic experience and were tracked via online surveys from a pre-experience baseline to four weeks post-use. Self-reported measures of well-being were collected one week before, and two and four weeks after psychedelic use. Acute subjective drug effects, dosage and contextual variables pertaining to the setting of use were measured on the day after the session. Repeated-measures analyses of covariance, *t*- and *z*-tests, as well as exploratory correlational and regression analyses tested differences in psychological changes, acute drug effects, and side effects between the two groups. Psychological well-being significantly improved in adolescents two and four weeks following psychedelic use, with a clinically relevant mean change score of 3.3 points (95% CI: 1.1–5.5). on the Warwick-Edinburgh Mental Wellbeing Scale [*F*(1.8, 172.9) = 13.41, *η*^2^G = .04, *p* < .001], statistically indistinguishable from changes in adults. Acute subjective effects differed between the age groups; adolescents reported significantly higher challenging experiences and ego-dissolution. In adolescents, visual symptoms related to “hallucinogen persisting perceptual disorder” (HPPD) were reported at a higher prevalence than in adults (73.5% vs. 34.2%, *p* < .001) but were reported as distressing by only one adolescent participant. To our knowledge, this is the first prospective study to examine the psychological effects of psychedelic use specifically in adolescents. Statistically significant improvements in psychological well-being and other domains of mental health were observed, consistent with effects seen previously in adults, providing tentative evidence for the potential utility of psychedelic interventions in adolescents. However, differences in acute subjective effects, specifically the less positive role of ego-dissolution experiences for long-term changes in adolescents, as well as a higher prevalence of HPPD-related symptoms suggest that special considerations might be required when assessing psychedelic treatment design and risks.

## 1 Introduction

Mental health problems remain one of the leading causes of disability worldwide, with more than half of all lifetime mental illnesses beginning during adolescence (1, 2). The definition of adolescence can vary between fields of study with slightly different theories and frameworks characterising adolescence. Within developmental psychology, adolescence is theorised by Erikson (3) as a time during which identity becomes the focus of concern, hypothesised roughly between ages 12–18, whilst Arnett (4) proposed a period of late teens/emerging adulthood encompassing ages 18–25. From a neurobiological perspective, adolescence is a crucial period of neurodevelopmental plasticity between the ages 10–24 (5–8), characterised by significant structural and functional changes in the brain. Whilst the limbic and reward systems and pubertal processes fully mature by mid-adolescence, key areas such as the pre-frontal cortex (involved in decision-making, impulse control, planning, and emotional regulation) take several more years to fully develop, continuing beyond the age of 20 (9–11). Taken together, adolescence will refer to the large span of ages 10 through 24 for the current study, divided into early (10–14), middle (15–17) and late (18–24) adolescence.

Given the highly malleable adolescent brain (12–14) and the developmental imbalance between puberty, behaviour, cognition, and emotion, this leads to heightened vulnerability to cognitive developmental changes and risk-taking behaviours, leaving “windows of opportunity” for the rise of mental health problems (15), as well as psychopathological symptoms such as suicidal thoughts and behaviours which is known to contribute to global youth disability (16). This further gives rise to the pressing need for adolescent mental health interventions. The implementation of interventions to improve adolescent mental health and psychological well-being (17–19) is considered to have a large impact across a range of health and psychosocial domains into adulthood (20). Its foundational role for adult health, it has been argued, warrants adolescent mental health to be integrated into public health policies (21), and research into novel interventions appropriate for adolescents to deserve prioritisation (22, 23). With mental health problems often left undiagnosed or untreated until early adulthood (18–24 years), poorer adult health becomes a likely if not inevitable consequence (2, 24).

Serotonergic psychedelics such as psilocybin have recently re-emerged into the research landscape as promising experimental medicines for the treatment of mental illnesses (25). Such mental illnesses are known to be prevalent in adolescents, such as depression and anxiety disorders (26, 27). Mostly small-scale, controlled, clinical studies on psychedelic use usually limited to adult populations have been performed to date (28), and only a few conducted with a relatively large sample size (29). In these studies, rapid and enduring improvements in mental health outcomes have been observed in various domains, including depression (25, 30–32), anxiety (30, 33, 34), alcohol use disorder (35) and symptoms of obsessive-compulsive disorder (36).

Healthy individuals taking psychedelics have also shown long-term improvements in positive domains of psychological wellbeing and functioning in controlled (37, 38) and naturalistic settings (39–42), which included younger populations aged between 18 and 24. These studies approach wellbeing as a broad, multidimensional construct constituting both feeling good (“being well”) or *hedonia* (43), and functioning well (“staying well”) or *eudaimonia* (44–47). Specifically, Mans et al. (42) found that following psychedelic use, participants showed increases in “being well” measures i.e., emotional stability, self-esteem, as well as “staying well” measures i.e., mindfulness, resilience, psychological flexibility, experiential acceptance, and social connectedness, except spirituality and compassion. Another key component which presented long-term psychological improvements following psychedelic use is connectedness, which is the sense of connection to oneself, others, and the universe (48, 49). Importantly, these positive wellbeing constructs are also known to be protective of adolescent mental health (50–58). Furthermore, large cross-sectional population studies which included participants aged 18 and over showed that following psychedelic use, rates of psychological distress and suicidality were reduced (59, 60), and that psilocybin use was associated with lowered odds of suicidal thoughts in (61). Taken together, the accumulating evidence has led some to argue for a prophylactic potential of psychedelics (23), although their longitudinal effects across different stages of development have never been tested in humans and confirmation and selection biases in prior studies should temper excessively enthusiastic extrapolations.

Overall, controlled research on the effects of psychedelics before adulthood is extremely scarce, with only a small number of early studies reporting beneficial effects of LSD and psilocybin on children and adolescents aged between 8 and 18 years old with autism and schizophrenia diagnoses (e.g., improvements in symptoms such as hallucinations and mood disturbances, and enhanced communication skills and social functioning) (62–65). These studies are limited however, by the use of outcome measures that lack the validity of contemporary research standards and were often less rigorous in methodology. More recently, a cross-sectional sectional study of adolescents (ages 16.52 ± 1.34) who had used ayahuasca within the context of religious groups in Brazil showed no signs of neuropsychological abnormalities while presenting fewer psychiatric symptoms compared with age-matched controls (66–68). However, the lack of a prospective within-subject component and potentially confounding factors associated with religious affiliation pose limitations on the inferences that can be drawn from these cross-sectional studies. Hence, although psychedelic use remains most prevalent amongst adolescents (69), with past-year LSD-use having increased by more than two-fold between 2005 and 2015 [from 1.22%–3.37% in US Americans aged 18–25 years; Killion et al. (70)], the specific potential benefits and risks of psychedelic use in adolescent demographics remains largely unstudied.

In humans, commonly used so-called “classic” psychedelics such as LSD or psilocybin are considered to have low potential for dependence (71–74), low physiological toxicity (73) even in long-term users (68, 75) and a positive acute tolerability profile when used under favourable extra pharmacological conditions, i.e., ensuring a good “set and setting” (76). Nevertheless, there exist several categories of acute and long-term risks associated with psychedelic use, which deserve special consideration in the case of vulnerable populations such as adolescents. One such risk involves the notion that psychedelic use could trigger prolonged psychotic symptoms and potentially even chronic psychotic disorders (77, 78). The peak age of onset for psychotic disorders is within late adolescence (79, 80) and the use of other psychoactive substances, such as cannabis, during adolescence, has been associated with an elevated risk of developing psychotic disorders (81). Thus, it is conceivable that psychedelic use could elevate the risk of psychosis in adolescents. Indeed, a longitudinal community sample of 2,588 German adolescents showed a 2.37 times elevated likelihood of experiencing psychotic symptoms in those who had used psychedelics five or more times, assessed at a 10-year follow-up, even after adjusting for other psychosis risk factors (82).

Another more commonly reported adverse long-term effect of psychedelic use lies in the occurrence of enduring perceptual abnormalities, particularly in the visual domain (83, 84). When visual effects such as “trailing”, intensification of colour, or “visual snow” persist after psychedelic use and are either perceived as distressing or lead to functional impairment, they are clinically recognised as “hallucinogen persisting perceptual disorder” (85). Survey studies indicate that while the occurrence of persisting visual aberrations might be as high as in 40% of users (86), they are in most cases sufficiently mild or transient to not be perceived as distressing, thus keeping the estimated prevalence of HPPD low at <1% (86–89). While the pathophysiological mechanisms underlying HPPD are yet to be uncovered (90), it is plausible that elevated neuroplastic changes in adolescence combined with the likely neuroplastic effects of psychedelics may put adolescents at special risk of developing the disorder. For example, a prospective online cohort study identified younger age as a significant predictor of HPPD-like symptoms following psychedelic use (89), however, this finding may be limited by its observational nature.

Lastly, perhaps the most immediate possible differentiator between risks to adults vs. adolescents via psychedelic-use lies in differences in the quality of the acute subjective experience. Previous research has reliably shown that the long-term psychological effects of psychedelics are moderated by the quality of acute subjective drug effects (91, 92). So-called “peak” (93) experiences deemed “mystical” (38), plus so-called “ego-dissolution” (94, 95), and emotional breakthrough experiences (96, 97) have been associated with more positive treatment effects, or improvements in wellbeing. Additionally, in group settings, the experience of “communitas” or an experience of shared of community or shared identity has been found to moderate longer-term psychological changes (98–100). Conversely, experiences of anxiety, dysphoria, paranoia, or confusion, not uncommon in response to psychedelic intoxication, and commonly grouped into “challenging experiences” (101), have in several studies, been found be negatively associated with subsequent outcomes (102–104). Here, too, the influence of contextual “set and setting” factors on the quality of the acute and sub-acute psychedelic experience is considered of critical importance (105–108) and has recently been demonstrated quantitatively (40, 42, 99, 109, 110). To our knowledge, no prior studies have investigated the relationship between set and setting, acute psychedelic effects, and long-term outcomes as a function of age, even though the greater susceptibility of adolescents to environmental influences (111, 112), and age-dependent differences in motives for substance use (113–115) suggest that adolescents may require different contexts than adults to produce optimal effects.

Overall, given that there is currently a public health risk due to the rising rates of naturalistic psychedelic use, with adolescents being particularly at risk (116), and that our knowledge on psychedelics on young populations undergoing a crucial developmental period are extremely limited, this further emphasises the relevance of our present study. The purpose of the present study was to compare the prospective effects of psychedelic use on psychological wellbeing and several mental-health related secondary outcomes in adults vs. adolescents. Additionally, we aimed to explore mechanistic specificities in the adolescent demographic, examining differences in acute subjective drug effects, their relationship with changes in wellbeing, and predictive contextual factors across both age groups. Lastly, adverse effects, including symptoms of psychosis and HPPD were analysed for adolescents and adults. To our knowledge, this is the first systematic study investigating psychological effects of psychedelics in an adolescent population that seeks to confirm whether adolescents and adults have differential acute experiences and long-term effects following psychedelic use.

## 2 Methods

### 2.1 Design

Data were pooled from two prospective cohort studies conducted between March and November 2017 [Cohort 1, presented in Haijen et al. (40)] and between April 2018 and June 2020 (Cohort 2) that were previously collected via an opportunity sampling approach. Participants could sign up to the study through a website (117), where they were notified about the study design and what was expected of them before signalling consent, and indicated the date of their planned psychedelic experience, which, importantly, was not endorsed in the study materials. Participants who gave consent were sent automated emails containing links to the surveys hosted on the surveygizmo online platform at multiple time points based on the indicated date of the experience.

**Figure 1:**
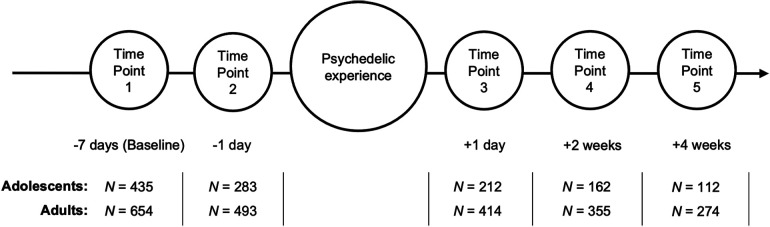
Survey study timeline. Circles represent the five time points of survey measurement, below each of which are the days in reference to the psychedelic experience. *N*: sample size of adolescent and adult participants at each time point.

Survey data on participants' psychedelic experiences and personal traits were collected anonymously in a non-controlled and observational manner using opportunity sampling and web-based data collection. A total of five surveys were given and completed at different time points: one week before the planned psychedelic experience (TP1); 3–24 h before the experience (TP2); one day after the experience (TP3); two weeks (TP4) and four weeks (TP5) after the experience (Figure 1). Only the methods and measures from this study relevant to the current study are presented here. For an overview of the full design, applicable to both Cohort studies, see Haijen et al. (40).

### 2.2 Participants and recruitment

 Participants were recruited through online advertisements shared on Facebook, Twitter, email newsletters and online drug-related public platforms (118, 119), including a link to the main website that hosted the survey. Recruitment criteria in both Cohort studies were: good understanding of the English language, at least 16 years of age and the intention to take LSD, N, N-dimethyltryptamine (DMT), ayahuasca, psilocybin/magic mushrooms/truffles, mescaline, iboga/ibogaine or hallucinogen-type NPS in the near future on their own initiative. Although adolescence is theorised to begin from age 10 at a neurobiological level (6) and age 12 coinciding with the beginning of identity formation (3, 120), adolescents between ages 10 and 15 were excluded from the recruitment criteria due to ethical and safety concerns, given their minority status (121, 122). For the current study, participants between ages 16 and 24 from Cohort 1 and 2 (Merged Cohort, *N* = 435) were included in the adolescent sample, while the sample size of participants aged 25 and older was *N* = 654 (Figure 1). A power analysis was not conducted due to the post-hoc and exploratory nature of the investigated hypotheses (123–125).

### 2.3 Measures

#### 2.3.1 Selection of measures

A total of 21 measures were selected for the current study, including measures covering general and broader aspects of well-being and mental health at baseline and endpoints; variables regarding the acute psychedelic experience; items relating to the “set” and “setting” prior to the experience; and side, as well as adverse effects during and after the experience. Only measures included in the present analyses are mentioned below, corresponding to the time point at which they were given. A summary of all measures used at each timepoint is shown in Supplementary Table S1.

#### 2.3.2 TP1 (baseline)

One week before their planned psychedelic experience, participants were asked to provide demographic information including age, sex, educational level, nationality, history of psychiatric illnesses, and previous use of psychedelic drugs and other substances i.e., non-psychedelic drugs and alcohol, using categorical responses ranging between Never and >100 times. Self-report information on whether participants considered themselves highly experienced drug users were collected to assess potential bias.

Eleven psychological outcome measures (outlined below) were selected for the current study, informed by a previous prospective study on psychedelic use using these measures (40, 42) as well as previous literature on adolescent well-being. Specifically, measures related to “being well” (hedonia) and “staying well” (eudaimonia) were selected, on the basis that the concept of well-being is a broad spectrum that is difficult to generalise while considering the social determinants of adolescent mental health (126).

The primary measure, the Warwick-Edinburgh Mental Wellbeing Scale (127) was used to evaluate psychological well-being and has been well-validated for use among those aged 16 years and over (128). Furthermore, the Quick Inventory of Depression Symptoms (129), also validated in adolescents aged 8–17 (130), was used to measure depressive symptoms, while the Rosenberg Self-Esteem Scale (131) was included as a measure of self-esteem. The Ten-Item Personality Inventory (132) was used to measure its subscale, “emotional stability”, which was validated in those aged 18–25 (133).

The Brief Resilience Scale (134) measured resilience, i.e., the ability to cope with and recover quickly from stress, while the Brief Experiential Avoidance Questionnaire (135) was used to measure experiential avoidance, i.e., the attempt to suppress uncomfortable internal experiences. Next, the Social Connectedness Scale (136) and the Watts Connectedness Scale (WCS) were used to measure social connectedness, and connectedness to self, others, and the world in general, respectively. Mindfulness was measured by the revised Cognitive and Affective Mindfulness Scale (137), while compassion was measured by the Santa Clara Brief Compassion Scale (138). Measures which assessed negative outcomes included the Suicidal Ideation Attributes Scale (139), validated in those aged 14–24 years (140), measuring suicidal ideation, the short version of the Spielberger State-Trait Anxiety Inventory (141) validated in those aged 12–21 years (142, 143), measuring trait anxiety, and Peter's Delusional Inventory (144) which was used to assess proneness to psychosis, measuring distress, preoccupation and conviction regarding delusional ideas.

#### 2.3.3 TP2 (pre-state)

The second survey was completed by participants between 3 and 24 h before the experience in order to obtain reports on the “set” of the individual right before taking the psychedelic substance (40). Among these, items under the short-form, self-constructed Psychedelic Predictor Scale (PPS) currently in preprint (145) measuring readiness and rapport were included in this study, with possible scores between 0 and 100 rated on Visual Analogue Scales.

#### 2.3.4 TP3 (post-acute psychedelic experience)

A day after the psychedelic experience, participants were asked what dose of the psychedelic drug was used based on typical LSD dose-equivalents in order to standardise dose measurements across non-LSD classical psychedelics (146). The options ranged from a “low dose” (no more than half a tab/50 micrograms of LSD) to an “extremely high dose” (more than 300 micrograms of LSD), split into five non-overlapping intervals.

The remainder of the third survey included measures evaluating acute subjective effects of the drug. The Mystical Experience Questionnaire (147) measured aspects relating to mystical experiences, while the Challenging Experience Questionnaire (148) evaluated challenging experiences and provided total as well as subscores for seven factors: grief, fear, death, insanity, isolation, physical distress, and paranoia. Emotional breakthrough, i.e., emotional release from overcoming difficulty, was assessed using the Emotional Breakthrough Inventory (149), while ego-dissolution, i.e., a loss of one's usual sense of self, was measured by the Ego-Dissolution Inventory (146).

Furthermore, the following items relating to the “setting” of the psychedelic experience were asked: “In what environment did the majority of your psychedelic experience take place?”, “Did your experience take place within a psychedelic drug retreat?”, “Was the setting designed and/or prepared with a therapeutic objective in mind?”, and “Was the setting more designed and/or suited for a recreational and/or social occasion, such as a party?”. Participants then indicated the number of people who were present for much of their experience, and whether there were individuals present who looked after them throughout. Lastly, the survey asked about physical side effects during the acute psychedelic experience.

#### 2.3.5 TP4 & 5 (two and four weeks later)

The remaining two surveys were given two and four weeks after the planned date of their psychedelic experience respectively and contained the same measures from the first survey to obtain follow-up responses. For Cohort 1 specifically, the fifth survey also contained an investigator-constructed self-rated measure of Hallucinogen Persisting Perception Disorder (HPPD) (150) symptoms according to DSM-5 criteria, with the question as follows: “Do you re-experience one or more of the following symptoms after cessation of use of a hallucinogen? Check the box(es) that apply: geometric hallucinations, false perceptions of movement in peripheral and visual fields, flashes of colours, intensified colours, trails of images of moving objects, positive afterimages, halos around objects, macropsia, micropsia, or none of the above.” And secondly “Do these cause significant distress or impairment in social, occupational, or other important areas of functioning?”. Cohort 2 had a different focus hence the HPPD measure was not collected.

### 2.4 Statistical analyses

#### 2.4.1 Pre- to post-psychedelic changes in well-being and secondary outcomes

Repeated measures analysis of variances (RM ANOVAs) with a Huynh-Feldt correction were conducted to assess the changes in well-being scores over time and were favoured over multivariate ANOVA (MANOVA) due to the large inconsistency in sample size of cohort-specific measures. Moreover, multiple RM ANOVAs are better at handling dependent variables that are highly positively correlated (*r*_s _> .06) or not significantly related at all (151, 152). Multiple comparisons of well-being scores across all three timepoints were corrected with Bonferroni adjustment post-hoc. Non-normally distributed data were also analysed using RM ANOVAs given the robustness of ANOVAs to normality violations (153, 154). The first RM ANOVA included as the primary measure of psychological well-being, WEMWBS as the dependent variable within the adolescent sample, with time as the within-subjects effect. WEMWBS was then assessed between age groups (adolescents vs. adults) using an RM Mixed ANCOVA (analysis of covariance), additionally including age group as the between-subject effect and above identified confounding variables as covariates. Further RM ANOVAs were conducted to assess changes on secondary outcome measures within the adolescent sample, including depression (QIDS), emotional stability (TIPI-ES), resilience (BRS), self-esteem (RSE), compassion (SCBCS), mindfulness (CAMS-R), experiential avoidance (BEA-Q), connectedness (WCS), social connectedness (SCS), suicidal ideation (SIDAS), and delusional thinking (PDI). Cases were excluded listwise, hence analyses only included responses completed for all three time points.

#### 2.4.2 Correlation between lifetime psychedelic use and baseline wellbeing

Two-tailed Spearman correlation tests were conducted to evaluate the association between baseline well-being measures and previous psychedelic use in adolescents.

#### 2.4.3 Identification of confounders

To identify differences in potentially confounding variables between adults and adolescents for the following analyses, Pearson's Chi-Square tests were performed comparing both age groups on the variables: gender, frequency of lifetime psychedelic use, other drug use (lifetime and past 6 months), history of psychiatric diagnoses (present or absent), and the bias item “I am a highly experienced psychedelic drug user”. Additionally, unpaired *t*-tests and nonparametric Wilcoxon rank-sum tests were conducted between the age groups on the psychedelic predictor scale (PPS) subscales “rapport” and “readiness”, and the following setting variables: being in a festival/club/party environment, being in psychedelic drug retreat, being in a therapeutic setting, being in a recreational/social setting, the number of people present during the experience, familiarity of the social environment, presence of a guide, and drug dose. Statistically different variables were included in downstream comparisons between adults and adolescents, after exclusion of any very strongly (*r* > 0.8) correlated pairs of variables in order to avoid multicollinearity issues.

#### 2.4.4 Relationship between acute subjective effects and extent of well-being change

A multiple linear regression model was constructed, comprising Z-standardised WEMWBS changes scores (difference in scores at baseline and at four weeks) as the dependent variable, while the following were included in the fixed part: age group (adolescent vs. adult) and its interactions with MEQ, CEQ, EDI, EBI, as well as the control variables baseline trait anxiety, the setting variables being in a psychedelic retreat and drug dose, and confounding variables. Two-tailed Pearson correlations were performed between the predictor and dependent variables to further confirm the findings from both models. To further corroborate differences in predictors of well-being changes, respective Pearson correlations were calculated for WEMWS change scores and acute experience measures that interacted with age group. Fisher's *z* tests were then performed to assess differences in correlation coefficients between the adolescent and adult samples.

#### 2.4.5 Differences in acute drug effects

Unpaired two-samples *t*-tests were performed between the age groups for mystical experiences (MEQ), challenging experiences (CEQ), ego-dissolution (EDI) and emotional breakthrough (EBI), as well as scores for all seven CEQ subscales, fear, grief, physical distress, insanity, isolation, death, and paranoia.

#### 2.4.6 Predicting adolescent-adult differences in acute experience scores

A multiple linear regression model was constructed to assess whether differences in acute experience measure scores previously found between adolescents and adults were merely a consequence of differences in setting and other confounding variables. Total scores on the acute experience measures, which showed a significant difference in previous *t*-tests, were included as dependent variables. Fixed variables included: age group (adolescents vs. adults), the setting variables being in psychedelic drug retreat and drug dose, as well as sex, baseline trait anxiety, and potential confounding variables. The CEQ subscales that were found to be significant in previous *t*-tests, were included as dependent variables in a similarly constructed model.

#### 2.4.7 Adolescent-adult differences in quality of experience

Unpaired two-samples *t*-tests were performed between the age groups for acute mystical experiences (MEQ), challenging experiences (CEQ), ego-dissolution (EDI) and emotional breakthrough (EBI), as well as scores for all seven CEQ subscales, fear, grief, physical distress, insanity, isolation, death, and paranoia. Kruskal-Wallis chi-squared tests were also conducted to further confirm their significance.

#### 2.4.8 Persisting adverse effects

Two-proportions *z*-tests (also known as a chi-square test for equality of two proportions) were conducted to compare the relative proportion of reports of each HPPD symptom.

#### 2.4.9 Cut-offs and guidelines

The significance threshold was set to *p* < .05. The strength of correlation was interpreted based on the guidelines of *r* = .10, *r* = .30 and *r* = .50, representing small, medium, and large effect sizes respectively, while the guidelines for partial eta squared effect sizes were 0.01 (small), 0.06 (medium) and 0.14 (large) (155). To test for multicollinearity, a Variance Inflation Factor (VIF) > 4 was used in regression analyses. The Shapiro-Wilk test and Q-Q Plot was used to test for normality. All statistical analyses were conducted in R 3.6.3 (156).

## 3 Results

### 3.1 Demographics

**Figure 2:**
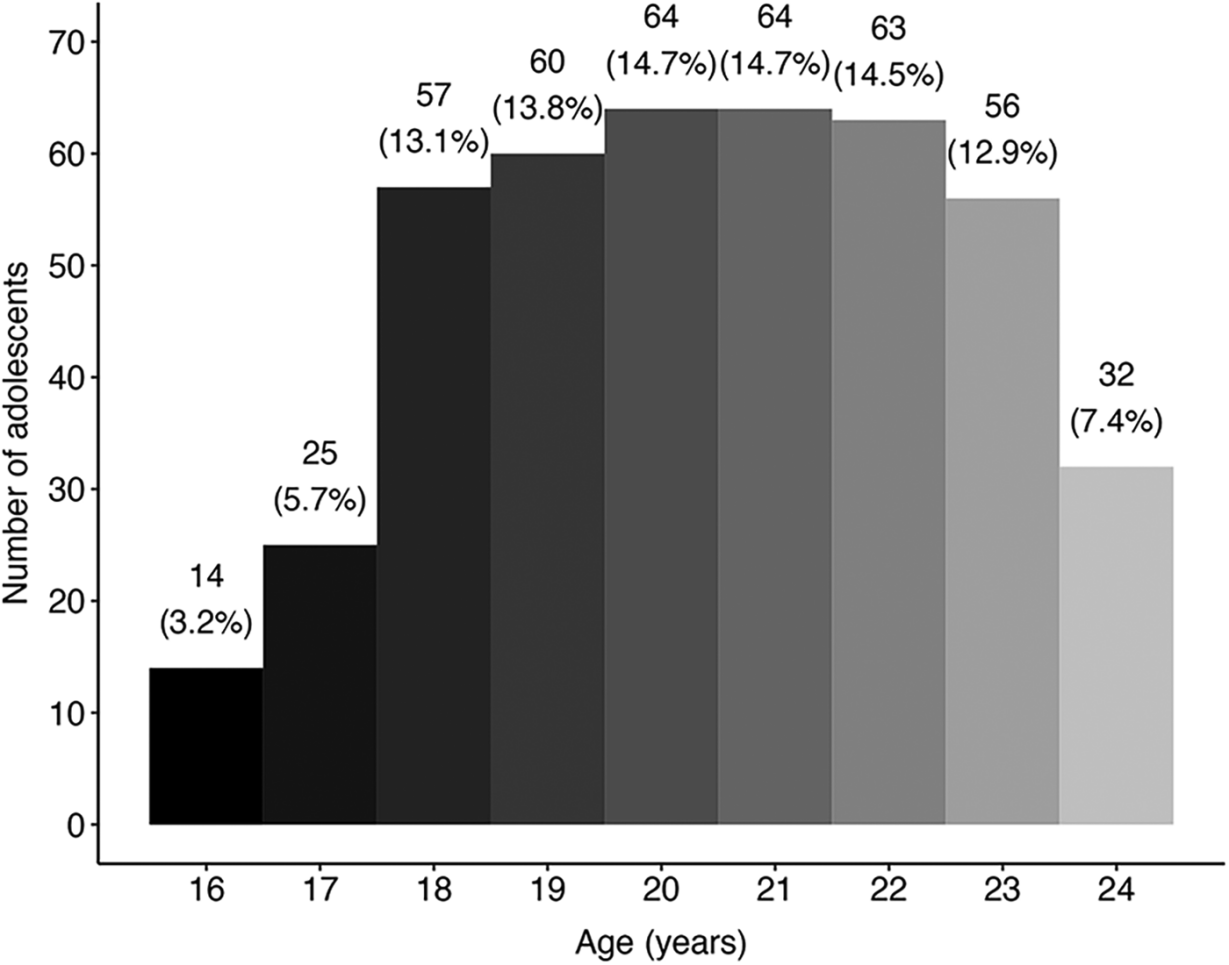
Frequency distribution of age within the merged adolescent sample. Binwidth = 1 year; *N* = 435.

Demographic information for baseline measures of the adolescent and adult populations within the two “opportunity” samples can be found in Table 1 (merged from Cohort 1 and Cohort 2; for cohort-specific breakdown see Supplementary Table S2), along with sample sizes at each time point. On average, adults were 37 years old (36.5 ± 9.7, *N* = 654) whilst adolescents were 20 years old (20.4 ± 2.2, *N* = 435) at baseline, with most of the adolescent sample aged 18–23 years old (Figure 2). In both age groups, a majority of the sample was male and had previous experience with psychedelics and other substances.

**Table 1:** Demographic data collected at TP1 (baseline) with sample sizes included at each time point.

Demographic		Adolescents	Adults
Sample size	TP1 (baseline; 1 week before)	435	654
	TP2 (3–24 h before)	283	493
	TP3 (1 day after)	212	414
	TP4 (2 weeks after)	162	355
	TP5 (4 weeks after)	112	274
Age		20.4 ± 2.2	36.5 ± 9.7
Gender	Male	331 (76.1%)	449 (68.7%)
	Female	100 (23.0%)	198 (30.3%)
	Other	4 (0.9%)	7 (1.1%)
Educational level	Left school before age 16 without qualifications	4 (0.9%)	16 (2.4%)
	High school/GCSE level (UK)	50 (11.5%)	21 (3.2%)
	High school diploma/A-Level (UK)	113 (25.9%)	50 (7.6%)
	Some university (or equivalent)	166 (38.1%)	116 (17.7%)
	Bachelor's degree (or equivalent)	92 (21.1%)	230 (35.2%)
	Post-graduate degree (e.g., Masters or Doctorate)	11 (2.5%)	221 (33.8%)
Nationality	United States	155 (35.6%)	151 (23.1%)
	United Kingdom	77 (17.7%)	166 (25.4%)
	Germany	31 (7.1%)	22 (3.4%)
	Canada	23 (5.3%)	40 (6.1%)
	Denmark	12 (2.8%)	52 (8.0%)
	Mexico	10 (2.3%)	4 (0.6%)
	Other (34 in total)	127 (29.2%)	219 (33.4%)
Psychiatric history	Have been diagnosed with at least one psychiatric illness in the past^a^	150 (34.5%)	254 (38.8%)
	Never been diagnosed with a psychiatric illness^a^	286 (65.5%)	400 (61.2%)
Other previous drug use	Other drugs—used at least once before^b^	421 (96.8%)	611 (93.4%)
	Other drugs—never used^b^	14 (3.2%)	43 (6.6%)
	Alcohol—consumer	303 (69.7%)	442 (67.6%)
	Alcohol—non-consumer	132 (30.3%)	212 (32.4%)

Absolute frequencies with corresponding percentages as well as means ± standard deviations are presented in the table.

^a^ Including major depressive disorder, bipolar disorder, anxiety disorder, schizophrenia, substance abuse disorder, alcohol dependence, hallucinogen persisting perception disorder, psychotic disorder, personality disorder, attention deficit hyperactivity disorder, obsessive compulsive disorder and/or eating disorder.

^b^ Including cannabis, amphetamine, MDMA/ecstasy, cocaine, opiates, benzodiazepines, and/or ketamine.

### 3.2 Changes in primary outcome well-being

**Figure 3:**
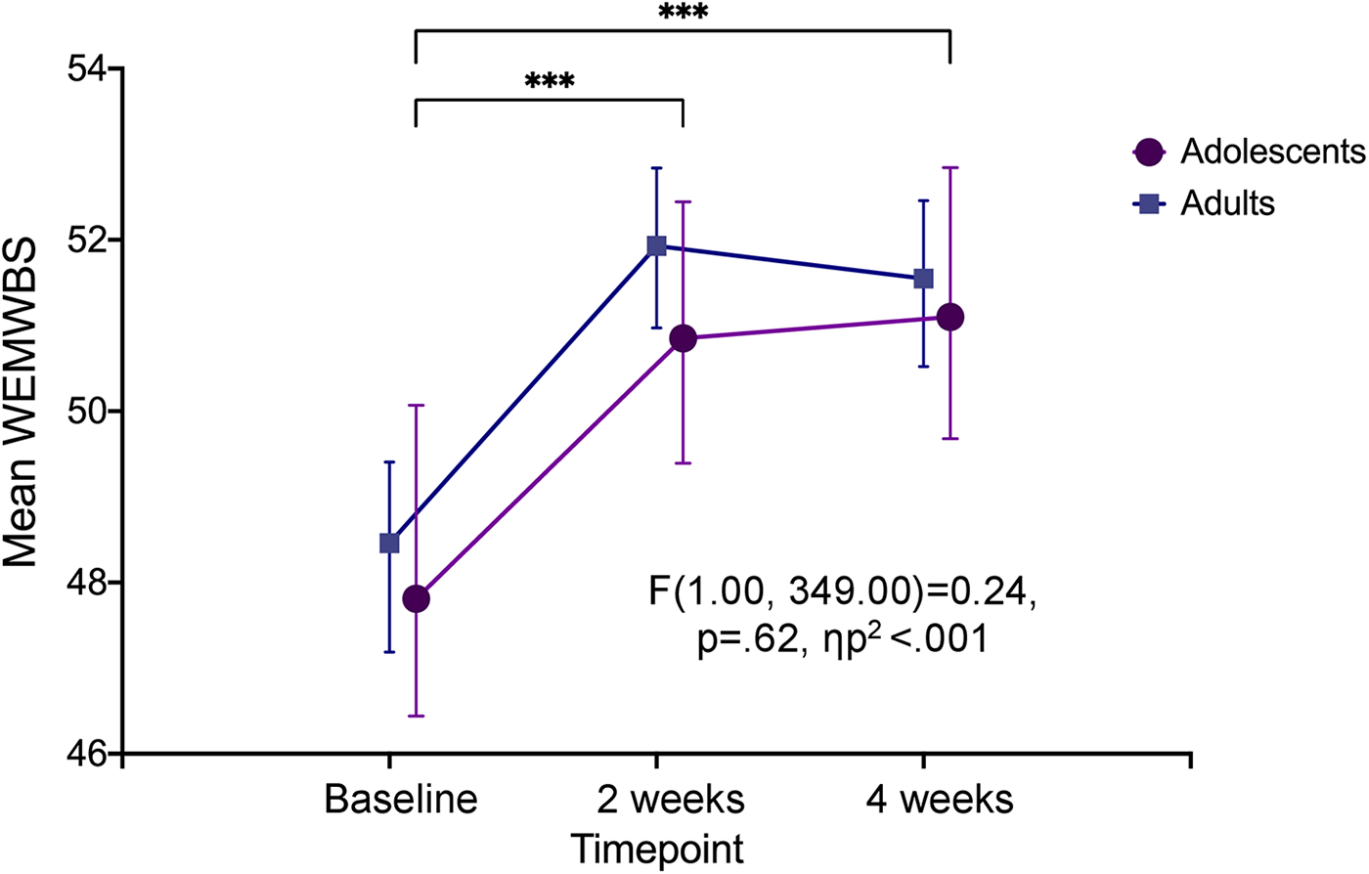
Psychological well-being at baseline, two and four weeks after the experience. Change in psychological well-being over three time-points seen within the adolescent sample (denoted by significance stars), and between the two age groups (*p* = .62). Error bars represent 95% confidence interval. ***p < .001. WEMWBS, Warwick-Edinburgh Mental Wellbeing Scale.

RM ANOVA analysis revealed that WEMWBS scores within the adolescent sample significantly differed between time points, *F*(1.8, 172.9) = 13.41, *p* < .001, *η*_p_^2 ^= .123. This was also seen for the adult sample, *F*(1.62, 412.65) = 35.82, *p* < .001, *η*_p_^2 ^= .123 (Table 2). The mean WEMWBS score for adolescents at baseline was 47.8 (95% CI: 46.1–49.5), and this significantly increased to 50.8 (95% CI: 49.4–52.3) at two weeks and 51.1 (95% CI: 49.7–52.5) at four weeks, hence a mean change score of 3.3 points (95% CI: 1.1–5.5). Meanwhile, adults displayed higher mean WEMWBS scores than adolescents at all three time points (Figure 3). Nevertheless, RM ANCOVA analysis with time as the within-subject effect revealed no significant interaction between time and age group (*p* = .48) and no difference in the between-subjects main effect of age group (*p* = .62; Table 2; Figure 3).

**Table 2:** WEMWBS means and corresponding statistics for RM mixed ANCOVA.

	Mauchly's test			RM Mixed ANCOVA				Mean (SD)			*N*
	Sig.	Correct.^a^	*ɛ*	*F*	df	Sig.	*η*p^2^	TP1	TP4	TP5	
Within-subjects					** **	** **					
Time	<.001	Yes	.84	17.07	1.70, 584.90	**<**.**001**	.**047**	48.29 (9.41)	51.63 (7.83)	51.43 (8.09)	353
Time** × **Age group	<.001	Yes	.84	0.68	1.69, 583.21	.48	.002	-	-	-	353
Between-subjects^b^					** **	** **					
Age group	<.001	Yes	.90	13.41	1.8, 172.9	**<**.**001**	.**123**	47.81 (8.56)	50.85 (7.48)	51.10 (6.96)	Adolescents (*N* = 97)
	<.001	Yes	.76	35.82	1.62, 412.65	**<**.**001**	.**123**	48.46 (9.73)	51.93 (7.96)	51.55 (8.49)	Adults (*N* = 256)

Significant values are in bold.

WEMWBS, Warwick-Edinburgh Mental Wellbeing Scale.

^a^ *ε* > .75 hence Huynh-Feldt correction was used (157).

^b^ Adolescent and adults both showed significant increases in WEMWBS scores over time respectively but did not significantly differ between each other in scores (*p* = .62).

### 3.3 Correlation between psychedelic-use frequency and baseline wellbeing in adolescents

**Figure 4:**
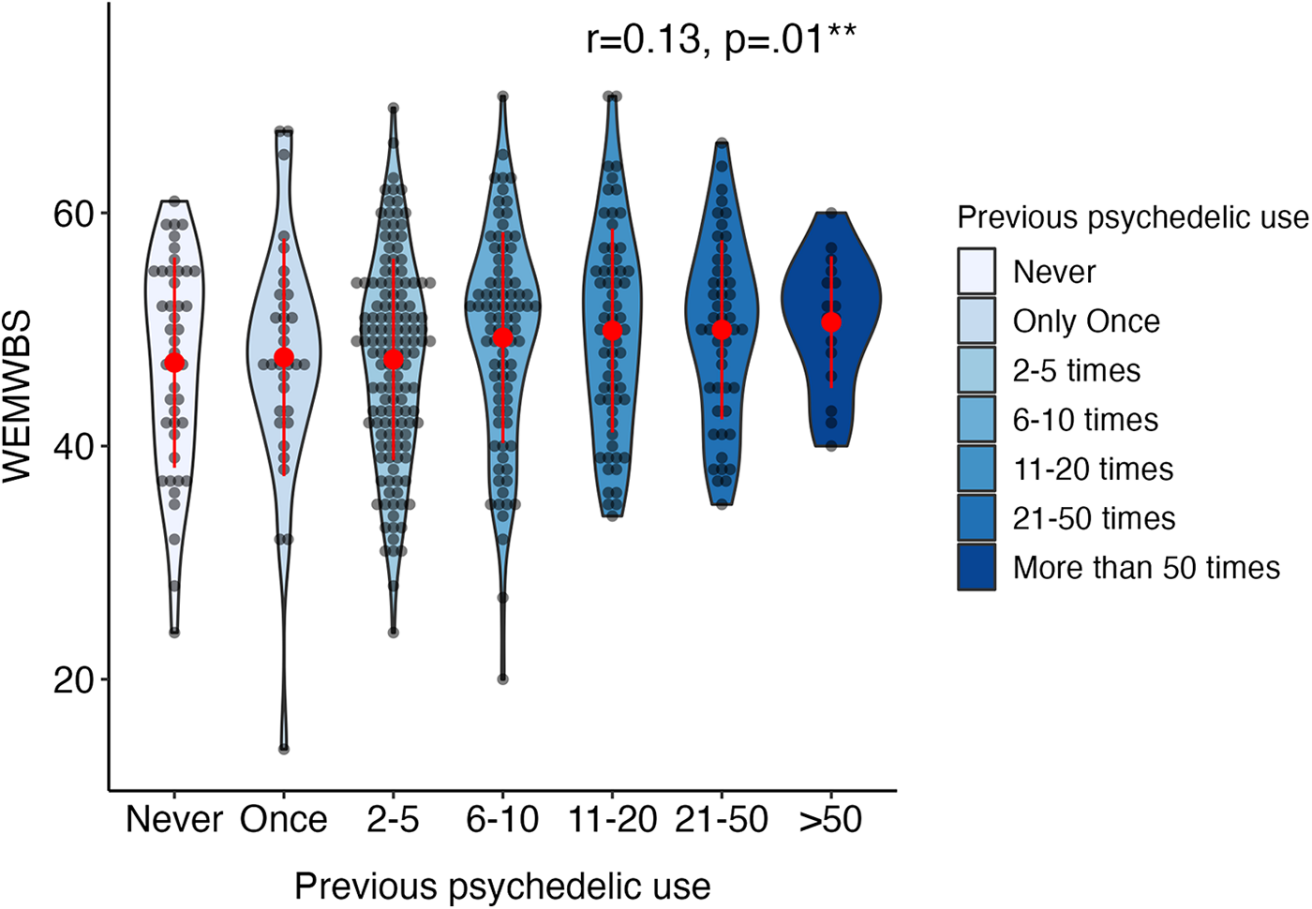
Distribution of statistically significant WEMWBS baseline measure in adolescents (aged 16–24) by frequency of previous psychedelic use. Width of violin plot denotes probability density in different values. Items “51–100 times” and “More than 100 times” are merged into “>50 times”. Red dots and lines represent mean and standard deviation (*N* = 435). Annotated in the plot are Spearman correlations between previous psychedelic use and the WEMWBS, Warwick-Edinburgh Mental Wellbeing Scale. *p < .05; **p < .01, ***p < .001.

Figure 4 illustrates the distribution of primary well-being measure WEMWBS at baseline by frequency of previous psychedelic use in adolescents. Psychological well-being showed a small but significant positive correlation with previous psychedelic use at baseline (*r* = .13, *p* = .01; see Supplementary Table S3).

### 3.4 Changes in secondary outcomes

**Figure 5:**
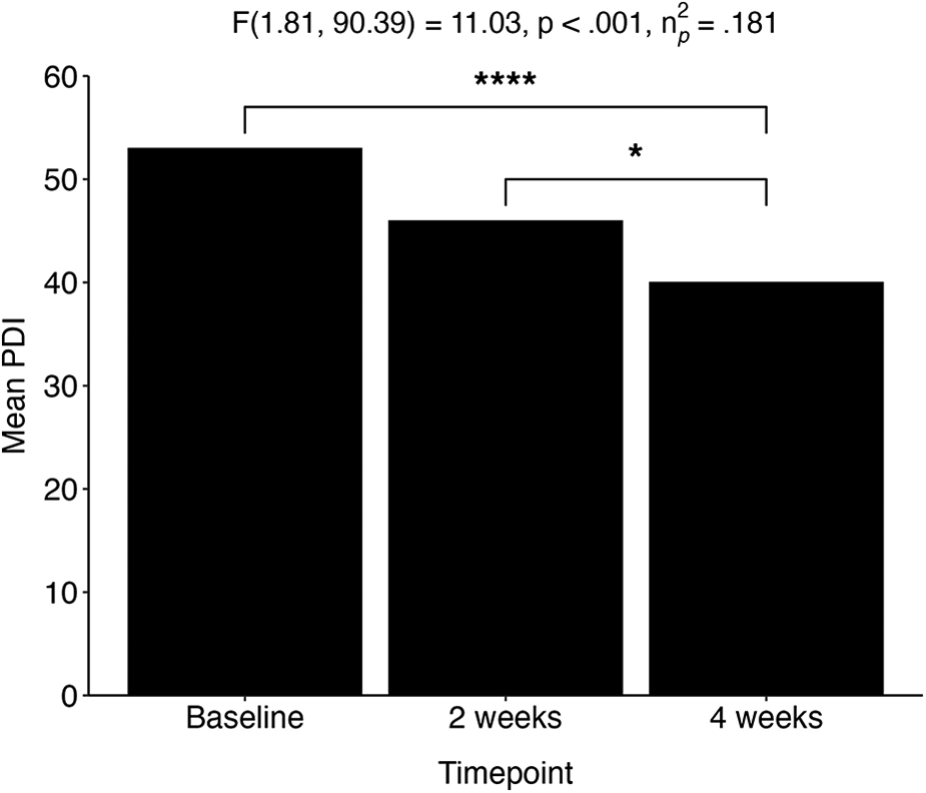
Adolescent population (aged 16–24). Change in delusional thinking over three time points: baseline, two weeks, and four weeks after the experience. *p < .05; ****p < .0001. PDI, Peter's Delusional Inventory.

Within the adolescent group, RM ANOVAs revealed a significant decrease over time for depression (QIDS) [*F*(1.59, 153.76) = 41.70, *p* < .001, *η*_p_^2 ^= .301], experiential avoidance (bEAQ) [*F*(1.52, 68.37) = 16.96, *p* < .001, *η*_p_^2^ = .274], suicidal ideation (SIDAS) [*X*^2^(1, *N* = 131) = 5.93, *p* = .02, *r* = .02,] and delusional ideation (PDI) [*F*(1.81, 90.39) = 11.03, *p* < .001, *η*_p_^2 ^= .181; Figure 5]. In addition, a significant increase over time was revealed for emotional stability (TIPI-ES) [*F*(1.00, 111) = 15.04, *p* < .001, *η*_p_^2 ^= .119], self-esteem (RSE) [*F*(1.75, 87.47) = 7.88, *p* < .001, *η*_p_^2 ^= .136], mindfulness (CAMS-R) [*F*(2,100) = 7.47, *p* < .001, *η*_p_^2 ^= .130], and connectedness (WCS) [*F*(1.62, 72.99) = 12.9, *p* < .001, *η*_p_^2 ^= .224] (Table 3). Post-hoc pairwise *t*-tests with Bonferroni correction showed that all measures changed significantly from baseline (*p* > .05). Additionally, no significant differences were observed in comparisons between baseline and two weeks for self-esteem (RSE), mindfulness (CAMS-R), and delusional thinking (PDI). The single pairwise comparison for suicidal ideation (SIDAS) and trait anxiety (STAI-T) between baseline and four weeks were initially significant but did not survive multiple comparison correction.

**Table 3:** Adolescents (aged 16–24): corresponding statistics for RM One-Way ANOVAs.

Measure	Mauchly's test			RM One-Way ANOVA				Mean (SD)			
	Sig.	Correct-ion^a^	*ɛ*	*F*	df	Sig	*η*_p_^2^	TP1	TP4	TP5	*N*
WEMWBS	<.001	Yes	.90	13.41	1.8, 172.9	**<** **.** **001**	**.** **123**	47.81 (8.56)	50.85 (7.48)	51.10 (6.96)	97
QIDS	<.001	Yes	.79	41.70	1.59, 153.76	**<** **.** **001**	**.** **301**	6.57 (3.93)	4.35 (2.88)	4.29 (3.12)	98
BEA-Q	<.001	Yes	.78	16.96	1.52, 68.37	**<** **.** **001**	**.** **274**	44.37 (11.67)	39.26 (9.89)	39.46 (9.93)	46
WCS	<.001	Yes	.81	12.97	1.62, 72.99	**<** **.** **001**	**.** **224**	56.55 (16.94)	63.86 (16.22)	64.58 (14.66)	46
PDI	.02	Yes	.90	11.03	1.81, 90.39	**<** **.** **001**	**.** **181**	53.04 (37.63)	46.02 (36.38)	40.06 (35.35)	51
RSE	.01	Yes	.88	7.88	1.75, 87.47	**<** **.** **001**	**.** **136**	18.29 (4.88)	19.82 (5.64)	20.20 (5.56)	51
CAMS-R	.12	No	-	7.47	2, 100	**<** **.** **001**	**.** **130**	31.26 (5.30)	32.37 (5.06)	33.28 (5.53)	51
TIPI-ES	-	No^b^	-	15.04	1.00, 111	**<** **.** **001**	**.** **119**	8.44 (3.28)	-	9.24 (3.05)	112
SIDAS	-	No^b^	-	9.55	1, 120	.002	.068	0 (4.50)	-	0 (2.00)	131
STAI-T	.02	No	.65	5.04	1.3, 177.5	.018	.035	43.2 (12.9)	48.0 (17.5)	45.6 (18.9)	138
BRS	-	No^b^	-	3.89	1.00, 127	.05	.030	3.23 (0.81)	-	3.43 (0.79)	128
SCS	.01	Yes	.89	1.13	1.79, 144.73	.35	.013	33.77 (10.20)	34.16 (11.10)	35.37 (9.90)	82
SCBCS	.17	No	-	0.01	2, 100	.99	<.001	23.31 (6.98)	23.35 (6.63)	23.29 (6.64)	51

Significant *p*-values are bold if surviving multiple comparisons. The actual *p* values listed above are before Bonferroni correction in *post-hoc* tests. Measures are ordered from highest to lowest partial effect size (*η*_p_^2^). SIDAS and STAI-T became non-significant after multiple comparison correction.

WEMWBS, Warwick-Edinburg Mental Wellbeing Scale; QIDS, Quick Inventory of Depression Symptoms; TIPI-ES, Ten-Item Personality Inventory-Emotional Stability; BRS, Brief Resilience Scale; RSE, Roseberg Self-Esteem Scale; SCBCS, Santa Clara Brief Compassion Scale; CAMS-R, Cognitive and Affective Mindfulness Scale; WCS, Watt's Connectedness Scale; SCS, Social Connectedness Scale; BEA-Q, Brief Experiential Avoidance Questionnaire; SIDAS, Suicidal Ideation Attributes Scale; PDI, Peter's Delusional Inventory; STAI-T, State-Trait Anxiety Inventory (Trait).

^a^ *ε* > .75 Huynh-Feldt correction was used (157).

^b^ Sphericity is assumed when there are two repeated measure factor levels, hence unnecessary to check TIPI-ES, BRS and SIDAS for violation (158).

### 3.5 Confounding variables

The adolescent and adult samples differed significantly for number of lifetime uses of a psychedelic [*X*^2^(12) = 139.56, *p* < .0001] and the bias item “I am a highly experienced psychedelic drug user” [*X*^2^ (4) = 18.58, *p* = .001]. Unsurprisingly, more adults (17.3%) were shown to have taken psychedelic drugs over 50 times in their lifetime compared with adolescents (3.7%), which complements the bias item demographic illustrating a significantly higher proportion of adults reporting as highly experienced drug users at baseline compared with adolescents (Supplementary Figure S1). Hence, previous psychedelic use and the bias item were included as confounding factors in further analyses.

Amongst all included variables related to set and setting, significant age-group differences were only observed for drug dose and being in a psychedelic drug retreat; adolescents were more likely to take higher doses of psychedelics than adults (*z* = −2.346, *p* = .019) while adults were more likely to have their psychedelic experience within the context of a psychedelic retreat compared with adolescents (*z* = −4.287, *p* < .0001; Table 4). Furthermore, scores on factors of the psychedelic predictor scale collected pre-state did not significantly differ between adolescents (*N* = 163) and adults (*N* = 286) throughout the experience, for both “rapport” (*p* = .318) and “readiness” subscales (*p* = .483). Only significantly different confounding variables between the age groups were included in further analyses.

**Table 4:** Descriptive data of confounding variables for adolescents and adults.

Confounding variables		Adolescents	Adults	Significance^c^
		*N* = 435	*N* = 654	
Previous psychedelic drug use^a^	Never (psychedelic-naïve)	44 (10.1%)	68 (10.4%)	**<** **.** **0001** **
	Once	34 (7.8%)	34 (5.2%)	
	2–5 times	138 (31.7%)	105 (16.1%)	
	6–10 times	87 (20.0%)	98 (15.0%)	
	11–20 times	65 (14.9%)	115 (17.6%)	
	21–50 times	51 (11.7%)	121 (18.5%)	
	More than 50 times^b^	16 (3.7%)	113 (17.3%)	
Bias item “I am a highly experienced psychedelic drug user”	Strongly disagree	54 (12.4%)	95 (14.5%)	**.** **001** *
	Disagree	126 (29.0%)	137 (20.9%)	
	Neither agree nor disagree	123 (28.3%)	155 (23.7%)	
	Agree	96 (22.1%)	182 (27.8%)	
	Strongly agree	36 (8.3%)	85 (13.0%)	
		*N* = 212	*N* = 414	
Drug dose^a^	Low	13 (6.1%)	53 (12.8%)	**.** **019** *
	Moderate	75 (35.4%)	155 (37.4%)	
	High	88 (41.5%)	136 (32.9%)	
	Very high	23 (10.8%)	47 (11.4%)	
	Extremely high	13 (6.1%)	23 (5.6%)	
Retreat setting	Yes	10 (4.7%)	67 (16.2%)	**<** **.** **0001** **
	No	202 (95.3%)	347 (83.8%)	

Absolute frequencies are shown along with corresponding percentage of absolute frequencies in brackets.

^a^ Including LSD, DMT/ayahuasca, psilocybin/magic mushrooms/truffles, mescaline (Peyote, San Pedro), Salvia Divinorum, Iboga/Ibogaine, and/or hallucinogen-type NPS.

^b^ Reported based on dose equivalents of the “reference-standard”, LSD: No more than 50 micrograms of LSD; No more than 100 micrograms of LSD; No more than 200 micrograms of LSD; No more than 300 micrograms of LSD; More than 300 micrograms of LSD.

^c^ In bold are significant *p*-values.

* *p* < .05.

** *p* < .001.

### 3.6 Predictors of well-being change

A multiple linear regression model was fitted with well-being change (WEMWBS scores at baseline—WEMWBS scores at four weeks) as the dependent variable, including the following covariates: measures of mystical experiences (MEQ), challenging experiences (CEQ), emotional breakthrough (EBI) and ego-dissolution (EDI), baseline scores of well-being and potential confounders: drug dose and being in a psychedelic drug retreat, bias item “I am a highly experienced psychedelic drug user” and previous psychedelic experience. The potential confounders did not show any significant influence on the model; hence, were excluded out of the restricted sample (Table 5).

**Table 5:** Regression model for WEMWBS change.

Fixed Effects	All Predictors		
	β*-*Estimate	95% CI	*p*-Value
Intercept	0.072	[−0.441, 0.586]	0.78
Age grp (Adol.)	−0.023	[−0.295, 0.249]	0.866
Retreat	−0.011	[−0.263, 0.239]	0.925
Dose	−0.09	[−0.207, 0.027]	0.132
Bias item	0.06	[−0.085, 0.205]	0.414
PPU	0.001	[−0.091, 0.095]	0.969
WB1	−0.572	[−0.705, −0.439]	**<** **.** **001** **
Age group × CEQ	0.144	[−0.127, 0.415]	0.295
Age group × MEQ	0.192	[−0.099, 0.484]!!!!	0.194
Age group × EBI	−0.204	[−0.511, 0.102]	0.19
Age group × EDI	−0.326	[−0.64, −0.011]	**0** **.** **042** *
Adj. *R*^2^	0.3494		
*F*-statistic	*F*(14,164) = 7.829, *p* < .0001		

CEQ, Challenging Experience Questionnaire; MEQ, Mystical Experience Questionnaire; EBI, Emotional Breakthrough Inventory; EDI, Ego Dissolution Inventory; Drug dose, LSD-equivalent drug dose; Bias item: “I am a highly experienced psychedelic drug user”; PPU, previous psychedelic use; WB1, baseline psychological well-being score. In bold are significant *p*-values.

* *p* < .05.

** *p* < .001.

**Figure 6:**
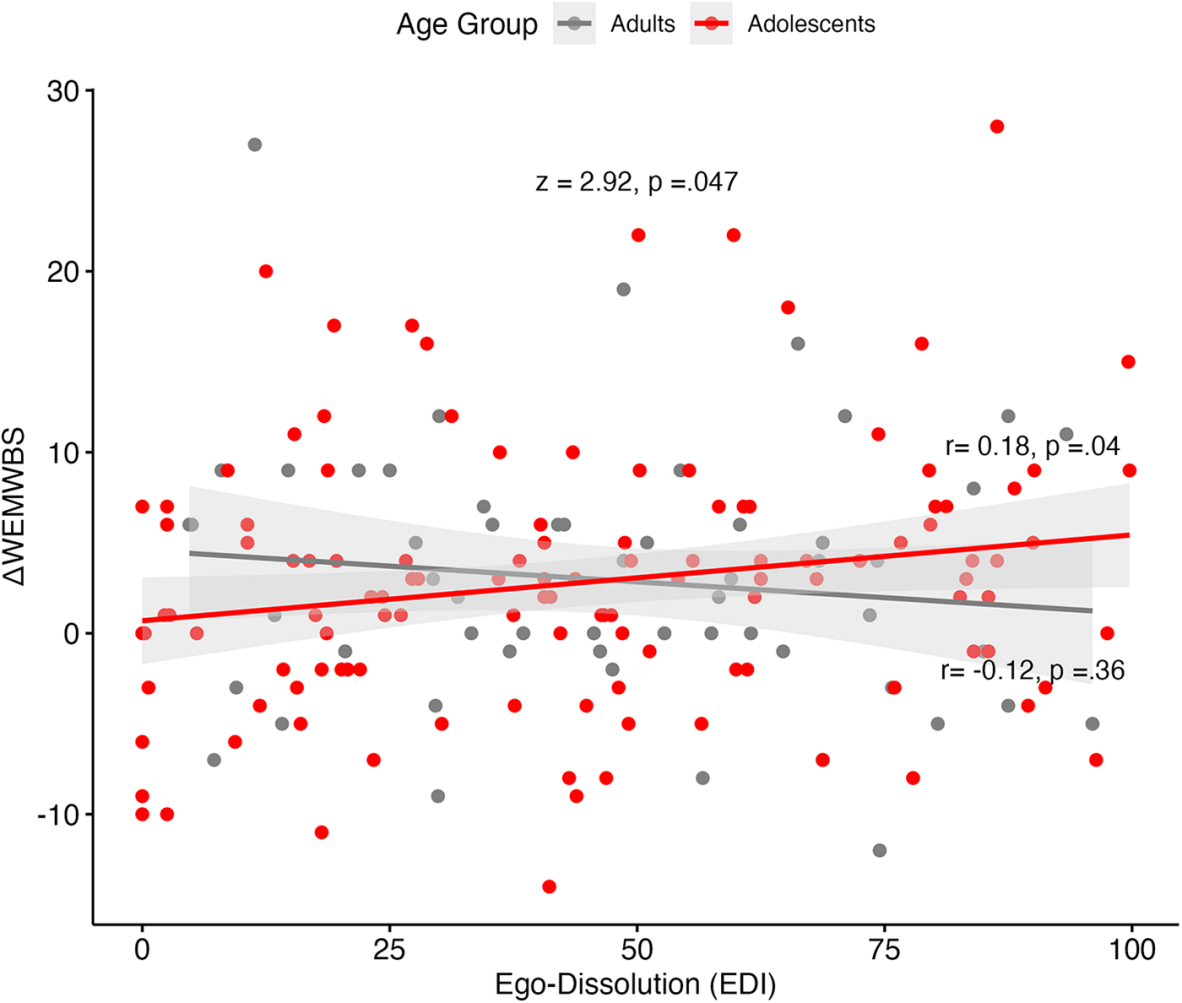
WEMWBS change scores against ego-dissolution (EDI) scores, by age group. Grey bandwidth = 95% confidence level interval for predictions from a linear model. Fischer's *z*-score presented in the graph, along with separate correlations of adult and adolescent samples. WEMWBS, Warwick-Edinburgh Mental Wellbeing Scale.

Controlling for acute experience measures, setting and baseline measures, adolescents did not significantly differ from adults in their extent of well-being change, reflected by the lack of predictive significance between age group and well-being change scores within the sample (*N* in analyses = 179; *p* = .866; Table 5). However, well-being change was significantly negatively predicted by the interaction between ego-dissolution and age group (*β *= −0.326, *p* = .042; Table 5), which remained significant after removing the main effects of acute experience measures. This was confirmed by Pearson correlations which showed a significant positive correlation between well-being change and EDI scores for adults (*r* = 0.18, *p* = .042), but not for adolescents (*r* = −0.12, *p* = .36; Figure 6). This difference in correlation strength was statistically significant (*z* = 2.92, *p* = .047; Figure 6).

### 3.7 Comparison of acute subjective effects between age groups

As displayed in Table 6, adolescents reported higher mean scores vs. adults for all four acute experience measures: mystical experiences (MEQ), challenging experiences (CEQ), ego-dissolution (EDI) and emotional breakthrough (EBI). However, only CEQ (*t* = 3.102, *p* < .001) and EDI (*t* = 1.852, *p* = .03) were statistically significantly different. Adolescents reported significantly higher mean scores for CEQ (22.3 ± 17.2; 95% CI: 20.97–23.6) compared with adults (18.0 ± 15.7; 95% CI: 16.8–19.2). For the EDI, adolescents reported a mean score of 44.4 ± 25.4 (95% CI: 40.6–47.9), while adults had a mean EDI score of 40.2 ± 28.8 (95% CI: 38.3–44.7; Table 6). Furthermore, exploratory follow-up tests for the seven CEQ subscales showed that all except “grief” (*p* = .40) and (experiencing one's) “death” (*p* = .88) were significantly higher for adolescents compared with adults, including “fear”, “physical distress”, “insanity”, “isolation”, and “paranoia”.

**Table 6:** Unpaired *t*-test results for acute experience measures between adults and adolescents.

Acute Experience Measures	df	Sig.	*t*	Cohen's *d*	Mean (SD)	
					Adolescents, *N* = 212	Adults, *N* = 414
MEQ	518	.14	1.464	0.136	42.0 (29.7)	37.7 (32.4)
EDI	624	.03*	1.852	0.150	44.4 (25.4)	40.2 (28.8)
EBI	624	.33	0.434	0.037	43.5 (30.0)	42.4 (32.7)
CEQ	624	<.001***	3.102	0.262	22.3 (17.2)	18.0 (15.7)
Fear	378	.002**	3.161	0.279	26.2 (25.2)	19.8 (22.0)
Grief	424	.31	0.510	0.043	23.3 (23.0)	22.3 (22.9)
Physical distress	624	.001**	3.058	0.258	25.2 (17.2)	20.6 (17.9)
Insanity	360	.001**	3.100	0.279	21.6 (26.6)	15.1 (21.8)
Isolation	380	.002**	3.033	0.267	24.3 (26.1)	17.9 (22.9)
Death	384	.710	0.372	0.033	12.0 (25.6)	11.2 (22.7)
Paranoia	318	<.0001***	3.370	0.319	10.4 (17.9)	5.80 (12.4)

MEQ, Mystical Experience questionnaire; EDI, Ego-dissolution Inventory; EBI, Emotional Breakthrough Inventory; CEQ, Challenging Experiences Questionnaire.

* *p* < .05.

** *p* < .01.

*** *p* < .001.

### 3.8 Predictors of acute drug effects

The fitted model included age group (adolescents vs. adults), confounding set and setting variables (drug dose and being in a psychedelic drug retreat), the item “I am a highly experienced psychedelic drug user” and previous psychedelic use, with challenging experiences (CEQ) and ego-dissolution (EDI) as dependent variables. Results showed that, across the entire sample (*N* in analysis = 576), when all other predictors were equal, age group (adolescents-adults) significantly predicted CEQ scores (*β *= 3.785, *p* = .007; Table 7). Meanwhile, an additional restricted sample excluding dose implied that higher doses may have accounted for the stronger reported ego-dissolution experiences in adolescents (Supplementary Table S4). This was reflected by a moderate, positive Spearman correlation between EDI and drug dose (*r*_S _= .032, *p* < .001). A similar multiple regression model was conducted for five out of the seven CEQ subscales: fear, physical distress, insanity, isolation, and paranoia. This was done as it was hypothesised that these subscales better reflect unpleasant challenging experiences. With all other predictors equal, a lower age group significantly predicted higher CEQ scores for all five subscales across the whole sample (*N* = 576; Supplementary Table S5).

**Table 7:** Regression model for ego-dissolution and challenging experiences.

	All Predictors		
	β*-*Estimate	95% CI	*p*-Value
Ego Dissolution (EDI)			
Fixed Effects			
Intercept	17.273	[9.168,25.377]	<.0001**
Age grp (Adol.)	3.147	[−1.602,7.896]	.194
Dose	6.591	[4.412,8.769]	<.0001**
Retreat (Yes)	7.056	[−0.077,14.188]	.053
Bias item	3.600	[1.108,6.093]	.005*
**Previous psychedelic use**	−1.518	[−3.17,0.134]	0.072
Adj. *R*^2^	0.07787		
*F*-statistic	*F*(5, 570) = 10.71, *p* < .0001		
Challenging Experiences (CEQ)			
Fixed Effects			
Intercept	15.189	[10.483,19.894]	.204
Age grp (Adol.)	3.785	[1.027,6.542]	.007*
Dose	3.225	[1.959,4.489]	<.0001**
Retreat (Yes)	9.754	[5.613,13.896]	<.0001**
Bias item	−2.121	[−3.567,−0.673]	.004*
**Previous psychedelic use**	−0.124	[−1.083,0.835]	0.799
Adj. *R*^2^	0.109		
*F*-statistic	*F*(5, 570) = 15.07, *p* < .0001		

Dose, LSD-equivalent drug dose; Retreat (Yes), being in a psychedelic drug retreat; Bias item, “I am a highly experienced psychedelic drug user”.

* *p* < .01.

** *p* < .001.

### 3.9 Differences in persisting adverse effects

**Figure 7:**
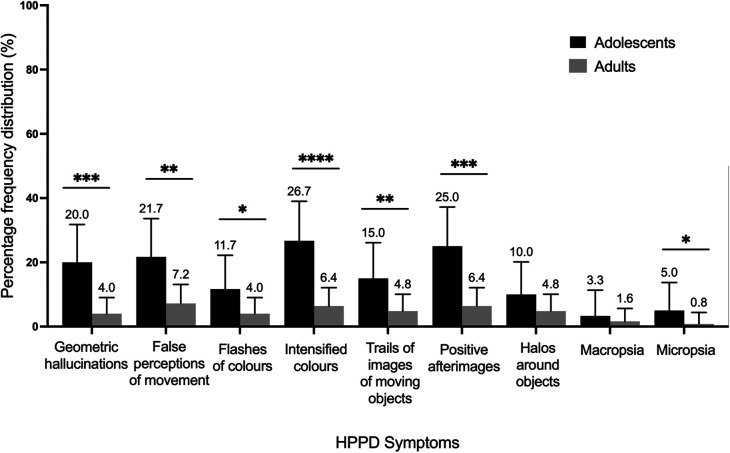
Relative percentage frequencies of HPPD symptoms reported by adolescents (*N* = 113) and adults (*N* = 168) four weeks after the experience. Error bars represent 95% confidence interval. *p < .05. HPPD, Hallucinogen Persisting Perception Disorder.

For Cohort 1, two-proportion *z*-tests on the HPPD measure taken at endpoint (at four weeks) revealed 22.4% of the adult sample (*N* = 28 out of 125) experienced any of the 9 listed symptoms (Figure 7), as opposed to 50.0% (*N* = 30 out of 60) of the adolescent sample (*p* < .001, *X*^2 ^= 13.1). Regarding specific HPPD-type symptoms, significant differences between the age groups were found for false movement perceptions (*p* < .001, *X*^2 ^= 8.1), colour flashes (*p* < .05, *X*^2 ^= 3.9), trails of images of moving objects (*p* < .01, *X*^2 ^= 5.7), micropsia (*p* < .05, *X*^2 ^= 3.4), intensified colours (*p* < .0001, *X*^2 ^= 14.7), geometric hallucinations (*p* < .001, *X*^2 ^= 12.4) and positive afterimages (*p* < .001, *X*^2 ^= 12.9; Figure 7). Importantly, only one participant in each age group responded affirmatively to the question of whether these symptoms caused them significant distress (<1%).

## 4 Discussion

The current study sought to investigate whether psychological responses to psychedelic compounds differ between adolescent and adults. Acute subjective effects, long-term psychological outcomes, and adverse side effects were all examined. Using a prospective online cohort study design, several convergences could be established between adult and adolescent psychedelic users, including equivalent improvements in psychological wellbeing and secondary measures related to mental health, such as depression, suicidality, self-esteem, and emotional stability. However, several distinguishing factors were also uncovered between adult and adolescent responses to psychedelics. Among these, differences in psychological mechanisms of change were observed between the age groups, where acute drug-induced experiences of ego-dissolution were less beneficial in adolescents than in adults. Psychologically challenging experiences were endorsed more strongly in the adolescents, partly due to higher doses used by the adolescent group. Furthermore, adverse long-term effects were more prevalent in adolescents, specifically visuoperceptual alterations related to HPPD, albeit not reported to be distressing. Summarizing, despite the overall favourable results, the ego-dissolution, challenging experience and HPPD data imply some elevated risk for psychedelic-use in populations under the age of 25.

### 4.1 Improvements in psychological well-being in adolescents post-experience

The here observed improvements in psychological well-being in adolescents following a psychedelic experience were consistent with effects seen in adults both in the current sample and in previous studies (31, 40, 42, 97, 159). At baseline, adolescent well-being scores were lower than normative data of the same age group in the UK population (mean = 51.7) (160, 161), but higher than that of a (non-normative) sample of US American students (mean = 46.5) (162), reflecting the multinational nature of the here analysed cohort. Effect sizes were slightly higher for adolescents, compared with adults, while the extent of change was not statistically different. Improvements in adolescents in other domains of mental health included decreases in suicidality and experiential avoidance, in line with previous findings in adults (163, 164). Depressive symptom severity, delusional ideation, connectedness, self-esteem, mindfulness, and emotional stability outcomes were also improved in adolescents. These findings are consistent with previously identified improvements in mindfulness (165–167), connectedness (48, 49, 99) and related variables (42) following psychedelic use in adults. Overall, the observed effects thus support the notion that psychedelics may have a prophylactic potential for mental health in young adults, as put forth recently (23). This further supports the idea that psychedelics, as a therapeutic intervention, can have significant implications in laying the groundwork for fostering resilience and coping skills in the developing adolescent brain, lasting into adulthood (8). This could have positive effects on long-term wellbeing outcomes in adolescents, such as reduced risk behaviours, improved academic performance, enhanced social relationships and overall quality of life (111).

While the present decrease in delusional ideation in adolescents challenges the historical but still influential notion of psychedelics as psychotogenic, these results should be viewed with caution given the potential of selection biases due to the observational nature of the study, as discussed below. With this limitation in mind, the finding does align with observed reductions in paranoid thinking in a recent psilocybin for depression trial (31), the absence of any significant changes in delusional ideation following LSD administration (78), and reduced symptoms of psychosis in psychedelic users in the general population (168). Although the observed effect size for decreases in delusional thinking in the current adolescent sample was relatively small, the findings add to these converging lines of evidence that speak against the notion that psychedelics are inherently psychotogenic—contrary to alarmist messaging of the past (169). We do, however, note one finding supportive of a psychotogenic potential with psychedelics (82).

### 4.2 First evidence of age-related differences in acute subjective effects of psychedelics

In line with previous findings showing that younger age is predictive of unpleasant reactions to psychedelics (109, 170), challenging experiences, most significantly experiences of paranoia, insanity, and fear, were significantly higher in adolescents than adults. This was the case even after controlling for confounding factors that differed between the age groups including higher drug doses used by adolescents, and the retreat context, which was more commonly reported by adults. Lacking education about the effects of psychedelics and related best practices might have contributed to the use of higher-than-average doses and more challenging experiences among the adolescents, pointing to the importance of psychoeducation for psychedelic harm-prevention approaches in young people.

Furthermore, and importantly, the relationship of ego-dissolution and wellbeing changes observed in the present study constitute the first, albeit preliminary, evidence of age-related differences in the mechanism of psychedelic-induced psychological changes. Specifically, the experience of ego-dissolution which is known to predict positive psychological changes in adults (94, 110, 146) had a significantly greater beneficial effect on well-being in adults compared with the adolescents, for whom this relationship was negative, albeit at a non-significant level. Intriguingly, this may suggest an age-related difference in the mediational role of ego-dissolution on subsequent key mental health outcomes. It is possible that for adolescents, ego-dissolution constitutes a more destabilising experience than for adults, perhaps due to lower ego-stability or higher basal ego-fragility at baseline (171). Future work could consider issuing an ego-stability inventory at screening or baseline to determine if it is predictive of subjective experiences (e.g., greater challenging experiences) or moderates the effects of ego-dissolution on mental health outcomes following psychedelic use. Differences in contexts of use could also be investigated as a moderating variable for the relationship between ego-dissolution and outcomes, considering that the presence of greater emotional support and post-experience integration are likely to help users derive benefit from intense experiences of ego-dissolution. Unstructured use, lacking frameworks for psychological preparation and integration may have been more prevalent amongst adolescent in the present sample, thus resulting in less positive long-term responses to experiences of acute ego-dissolution.

Considering the importance of the adolescent period for brain and mental health development, the possibility that psychedelic-induced ego-dissolution may destabilise self-development in a prolonged and potentially problematic way in some individuals, even if it is only a minority, warrants further controlled research. Disturbance-of-self is a common, if not fundamental component, of the incipient phase of psychotic disorders (172)—and the onset of such states or phases peaks during adolescence (173). While group-level reductions in delusional thinking in the adolescent population studied here are reassuring, it is possible that rare cases of iatrogenesis could have occurred but remained uncaptured due to selective study attrition, constituting a crucial limitation of the present observational study design.

### 4.3 Vulnerability to HPPD-like effects in adolescents after psychedelic use

Unexpectedly large differences were observed in reports of persisting visuoperceptual symptoms related to hallucinogen persisting perceptual disorder (HPPD) between adults and adolescents, the latter being more than twice as likely to report any persisting visual alterations at the four-week endpoint. Specifically, false movement perceptions, colour flashes, trails of images of moving objects, micropsia, intensified colours, geometric hallucinations and positive afterimages were reported by a significantly higher proportion of adolescents than adults at four weeks. Critically, these symptoms were perceived as distressing by only one adolescent and one adult participant (<1%), which likely explains the low diagnostic prevalence of HPPD despite seemingly frequent residual visual symptoms (174, 175). The observed difference in HPPD related symptoms between adolescents and adults may in part be explained by the reportedly higher doses taken by adolescents, although the magnitude of the difference suggests that adolescents may indeed be inherently more vulnerable to experiencing visual aftereffects following psychedelic use. On a neurophysiological level, the greater extent of HPPD-type symptoms in adolescents could be explained by the imbalance of excitatory and inhibitory synapses present during brain maturation (176). Computational studies have shown that HPPD could result from lacking inhibition, increasing excitation, or a combination of both in the primary visual cortex (177). The pre-existing excess of excitatory synapses over inhibitory ones in the visual cortex during adolescence (178) may thus predispose serotonin 2A (5-HT2A)-receptor-expressing inhibitory interneurons in this area of the brain to take damage for overexcitation, which remains the most likely theory of HPPD aetiology to date (179–181).

Taken together, these findings indicate that while adolescents may gain psychological benefit from psychedelic experiences similarly to adults, they tend to experience more difficulty when using psychedelics compared with older individuals, independently of setting, drug doses, or previous experience with psychedelics, and are more likely to develop HPPD-like effects after psychedelic use. These findings bear relevance to potential future psychedelic-assisted interventions for adolescents and should be taken into consideration when planning research with younger participants. The simplest implication is that younger participants or patients and their therapeutic providers should prepare for greater than average ego-dissolution and challenging experiences and establish good care and contingencies around this e.g., via aiming to build strong therapeutic relationships prior to the psychedelic experience—and to factor in good within-session supervision and post-session integration. These principles apply generally, but—according to the present study's results—may be especially important in adolescents. One line of research that has garnered greater urgency considering the present findings is the identification of protective measures which may prevent the development of persisting visual alterations following psychedelic use. A first hypothesis that should be tested in future research is whether the absence of external visual stimulation (e.g., by using eyeshades as is typically done during psychedelic clinical trials), might reduce the likelihood of developing HPPD-type effects. Moreover, although other previous drug use was not an age-related confounding factor at baseline in our study, future studies could investigate whether prohibiting the use of other substances throughout the duration of the study might also reduce HPPD-type effects, considering the theoretical reasons and past evidence, albeit anecdotal, on the potential interactions between psychedelics and other drugs such as cannabis, alcohol and psychostimulants (84, 90, 180, 182, 183).

### 4.4 Study strengths and limitations

The present study constitutes a significant step toward an ethical assessment of the feasibility of psychedelic-assisted interventions in adolescents. Age-related similarities and differences in response seen here serve to highlight our limited understanding of the differential effects of psychedelics during critical developmental periods. Much more research needs to be done before guidelines or policies can be recommended on this matter, other than to promote general principles of harm reduction with psychedelics, such as careful screening, dosing, and properly informed psychological support throughout. Special ethical considerations—such as dual consent (e.g., from a parent) for adolescents to receive a psychedelic may be worth considering in the future. Lastly, adolescent psychedelic users and study participants should be made aware of the possibility that they may be at greater risk of developing HPPD-like effects following psychedelic use.

This web-based observational study has some significant limitations. The lack of experimental control is an inherent shortcoming of observational studies. This includes the lack of drug dose verification. Subjective estimations of drug dose given retrospectively by participants may have biased observed relationships between dosage and key outcomes—such as ratings of ego-dissolution or challenging experience, for example.

There may be other important biases in these data. Firstly, the choice to omit power analysis due to the exploratory nature of the study could have given rise to higher generalisability. However, it can be argued that the primary aim was to explore relationships in our data taken from an already-existing dataset rather than test pre-defined hypotheses, and that due to the robustness of our sample sizes, the study has considerable statistical power to detect meaningful effects. Furthermore, the sample was restricted to those who had the intention of taking a psychedelic on their own initiative, hence it mainly comprised experienced psychedelic drug users. Most of the adolescent sample were moderate psychedelic users (2–5 lifetime uses), and thus not representative of the general population (the majority of whom are likely to be psychedelic-naïve) or indeed the adult population in this sample (who were relatively highly experienced with psychedelics). A high proportion of advocates of psychedelic use, well-versed in harm reduction and benefit maximisation strategies possibly due to recruitment through online drug-related public platforms could have skewed our findings in the direction of exaggerating benefits and deflating risks. Thus, our sample may be unrepresentative of those who take psychedelics in poorly planned or unintentional ways, and it is reasonable to suspect the risk of adverse reactions would be higher in such populations. This limits the generalisability of the present results to a wider population of psychedelic users. Additionally, the definition of “adolescence” is debatable as most definitions, including WHO (184), only go up to 19 years of age, although the ages 20–24 do fall within WHO's definition of “young people”. One could argue, however, for a modern, neuroscience informed definition of adolescence that would include the higher ages we have included here (7) which is supported by identity theory and neurobiological frameworks (3, 8), but we still acknowledge that our extended definition of adolescence could be criticized.

Further limiting generalisability of the results, a large proportion of participants were of white race and ethnicity and male. Although psychedelic-use in the USA is known to be more prevalent in males than females (168) and more prevalent in individuals of white race and ethnicity, as compared with other racial and ethnic groups (185), this bias did not allow for meaningful investigation of sex-, subethnic- or race-related differential responses to psychedelics (186).

The possibility of an attrition bias in the sample may have further skewed outcomes in a manner that exaggerates positive and diminishes negative effects. Previous work has shown that age is a good predictor of study dropout in one of the samples included here (187). Thus, many adolescents who signed up at baseline dropped out of the data pool at subsequent timepoints—and we did not collect any explanations from them for why they did so. Predictive analyses of drop-out, assessing such variables as high CEQ scores, baseline psychosis proneness, or worsening of mental health outcomes post psychedelic-use, did not suggest that drop-out was triggered by negative response (187).

Future studies should address these limitations to help improve the validity, generalisability, and inclusivity of the findings. Firstly, future research should include mans to minimize attrition (e.g., offering incentives for participation and maintaining regular contact) and collect feedback on reasons for dropout. Given the low number of female participants in this study, hypotheses should test whether removing female samples would affect the findings, or whether there are sex-related differential experiences to psychedelics. Studies should also expand to include participants with varying levels of psychedelic drug experience to help mitigate bias, as well as diversify its population representation across different races, ethnicities, and socioeconomic backgrounds. Future controlled, randomised, double-blind trials can also effectively overcome the inherent lack of control found in observational studies. Importantly, some theoretical definitions of adolescence highlight that adolescence begins earlier than 16 years old (3, 5, 188). It would be interesting to further understand the differential effects of psychedelics on populations at different developmental periods as defined by Erikson (3), namely: adolescence (12–24), young adulthood (25–39), middle adulthood (40–65) and late adulthood (beyond 65). Hence, the effects of psychedelics as a therapeutic intervention in even younger populations should be an important focus in future research, especially considering the complex ethical issues around paediatric psychedelic use (189).

Despite these limitations, the nature of the prospective study design used here, first implemented by Haijen et al. (40), has provided many advantages within psychedelic research—particularly in comparison with retrospective survey studies—where hindsight bias is a special issue. A major advantage of the prospective approach is its greater ability to support inferences about causal relationships and identify predictive factors within diverse, naturalistic samples. Multivariate analyses are possible on such data, allowing a larger number of questions to be asked than is typically possible in small, controlled studies. Moreover, naturalistic studies have an obvious advantage in terms of ecological validity over lab based controlled studies and are better able to collect large data pools, potentially allowing for the assessment of difficult to investigate populations, such as the one included here, as well as rare but important effects or events. Furthermore, missing data due to attrition was handled by excluding those participants from the final sample to avoid potential biases and enhance statistical power. Despite participants being removed, our study was able to conduct analyses on large sample sizes (*N* = 435 for adolescents and *N* = 654 for adults). Hence, the central limit theorem (190) applies in our study, allowing the sampling distribution of the parameters to be approximately normal even if the underlying population is non-normal. Our study handled non-normality through the large sample sizes and the robustness of ANOVAs, *t*-tests and multiple linear regressions to normality violations (153, 154, 191, 192), while maintaining validity and reliability of statistical analyses used.

## 5 Conclusion

To our knowledge, this is the first systematic study investigating acute and longer-term psychological effects of psychedelics in an adolescent population. Adolescence is a crucial period of brain and mind development. It is also a period of heightened vulnerability to the onset of mental illnesses that can be enduring and hugely costly (24, 193). Given the growing evidence for the therapeutic value of psychedelic therapy (122), the growing severity and/or prevalence of adolescent mental health issues (194, 195) which were further exacerbated during and after COVID-19 (27, 196, 197, 198), limitations in the effectiveness and feasibility of available adolescent-targeted treatments (199–204), high prevalence of psychedelic-use and gradual decline in the mean age of first exposure to psychedelics among younger populations (69, 205), and the growing awareness of the need for early, intervention in mental health care (206–209), it feels timely that we look more seriously at the potential value of psychedelic-assisted interventions in young people, while being mindful of potential risks. The present findings highlight the need for more research on this important topic.

## Data Availability

The raw data supporting the conclusions of this article will be made available by the authors, without undue reservation.
