# Supplementary material for: Psychological effects of psychedelics in adolescents
Source: Front Child Adolesc Psychiatry. 2024 Jun 7;3:1364617. doi: 10.3389/frcha.2024.1364617 (PMC11732004; doi:10.3389/frcha.2024.1364617)
Supplement: Supplementary file 1 [file Datasheet1.docx]

Supplementary Material

# Supplementary Figures

**

**Supplementary Figure 1**. One out of four items assessing positive attitudes towards psychedelic drugs grouped by age group. Item: “I am a highly experienced psychedelic drug user”. Adolescents, N=435; Adults, N=654. P-value denotes significance of Chi-Square test.

# Supplementary Tables

**Supplementary Table 1.** Summary of measures used at each time point (TP).

| No. | Measure | Constructs & (if present) subscale(s) used | Cronbach’s alpha | Was the measure validated on adolescents and young adults? | Timepoint | | | |
| --- | --- | --- | --- | --- | --- | --- | --- | --- |
|  |  |  |  |  | **TP1**  (Baseline) | **TP2**  (Pre-state) | **TP3**  (Post-acute psychedelic experience) | **TP4 & 5**  (Two and four weeks after experience) |
| 1 | **WEMWBS**  The Warwick-Edinburgh Mental Well-being scale (Tennant et al., 2007) | Psychological well-being | .91 | 16-24 years subgroup (Tennant et al. 2007) and (Clarke et al., 2011) | **✓** |  |  | **✓** |
| 2 | **QIDS**  Quick Inventory of  Depression Symptoms  (Rush et al., 2003) | Depression symptoms  All scales: sleep, feeling sad, appetite, concentration, sleep, view of oneself, suicide, interest, energy, psychomotor. | .86 | 8-17 years, Cronbach’s alpha 0.78 (Haley et al., 2023)  12-17 years, Cronbach’s alpha 0.77, (Zhang et al., 2020) | **✓** |  |  | **✓** |
| 3 | **RSE**  Rosenberg Self-Esteem  Scale (Rosenberg, 1965) | Self-esteem | .82 to .88 | 14-18 years (Rosenberg, 1965) | **✓** |  |  | **✓** |
| 4 | **TIPI**  Ten-item Personality  Inventory (Gosling,  Rentfrow, & Swann, 2003) | Personality  ⅕ subscales: “emotional stability” | .73 | 18-25 years, Cronbach’s alpha 0.83 (Atak, 2013) | **✓** |  |  | **✓** |
| 5 | **BEA-Q**  Brief Experiential Avoidance Questionnaire (Gámez et al., 2014) | Experiential avoidance | .80-.89 | 18-21 years subgroup (Gámez et al., 2014) | **✓** |  |  | **✓** |
| 6 | **BRS**  Brief Resilience Scale (Smith et al., 2008) | Resilience | .84-.87 | Mean age 20.4 and 19.8 years (Smith et al., 2008) | **✓** |  |  | **✓** |
| 7 | **SCBS**  Santa Clara Brief Compassion Scale (Hwang et al., 2008) | Compassion | .90 | Mean age 19.95 years (SD=1.33) (Hwang et al., 2008) | **✓** |  |  | **✓** |
| 8 | **CAMS-R**  Cognitive and Affective Mindfulness Scale–revised (Feldman, Hayes, Kumar, Greeson & Laurenceau, 2007) | Mindfulness | .76 | Mean age 19.31 years (SD = 2.66) (Feldman, Hayes, Kumar, Greeson & Laurenceau, 2007) | **✓** |  |  | **✓** |
| 9 | **WCS**  Watt’s Connectedness Scale (Watts et al. 2022) | Connectedness to self, others, and the world | .84 - .90 | Validated in people aged 16 and over in the Global Psychedelic Survey (psychedelicsurvey.com) | **✓** |  |  | **✓** |
| 10 | **SCS**  Social Connected Scale (Lee & Robbins, 1995) | Social connectedness | .91 | 14-18 years (Lee & Robbins, 1995) | **✓** |  |  | **✓** |
| 11 | **SIDAS**  Suicidal Ideation Attributes Scale (Spijker et al., 2014) | Suicidal ideation | .91 | 14-24 years, Cronbach’s alpha 0.83 (Gauvin et al., 2022) | **✓** |  |  | **✓** |
| 12 | **PDI**  Peter’s Delusional Inventory (Peters et al., 2004) | Delusional thinking | .90 | Mean age 19.1 years (SD = 2.5) (Peters et al., 2004) | **✓** |  |  | **✓** |
| 13 | **STAI-T**  Spielberger State-Trait Anxiety Inventory – short form, trait version (Marteau & Bekker, 1992) | Trait anxiety | .87 | 12-18 years (Garrido et al., 2022)  18-21 years, student sample (Maynard et al., 2010) | **✓** |  |  |  |
| 14 | **PPS**  Psychedelic Predictor Scale (Angyus et al., 2024) | Readiness and rapport | .80 | None (mean age in this study: 28.9 +/- 10.45) |  | **✓** |  |  |
| 15 | **Setting**  Items measuring variables regarding the setting of the psychedelic experience (self-constructed) | Setting | None, but settings questionnaire for ayahuasca show good reliability (Pontual et al., 2021) | None |  |  | **✓** |  |
| 16 | **Physical Side Effects**  (self-constructed) | Physical side effects post-experience | N/A | None |  |  | **✓** |  |
| 17 | **MEQ**  Mystical Experience Questionnaire (Barrett, Johnson & Griffiths, 2015) | Mystical-type experience | .97 | None |  |  | **✓** |  |
| 18 | **CEQ**  Challenging Experience Questionnaire (Barrett, Bradstreet, Leoutsakos, Johnson, & Griffiths, 2016) | Challenging experience | .80-.89 | None |  |  | **✓** |  |
| 19 | **EBI**  Emotional Breakthrough Inventory (Roseman et al., 2019) | Emotional breakthrough | .93 | None |  |  | **✓** |  |
| 20 | **EDI**  Ego-Dissolution Inventory (Nour, Evans, Nutt, & Carhart-Harris, 2016) | Ego dissolution | .93 | None |  |  | **✓** |  |
| 21 | **HPPD**  Hallucinogen persisting perception disorder – DSM-5 criteria | Symptoms of hallucinogen persisting perception disorder | .74 (based on Simonsson et al. (2023)) | None |  |  |  | **✓** |

**Supplementary Table 2**. Cohort-specific demographic data collected at Time Point 1 (baseline) and sample sizes at each timepoint.

|  | | **Cohort 1** | | | **Cohort 2** | |
| --- | --- | --- | --- | --- | --- | --- |
| **Demographic** | | **Adolescents** | **Adults** | **Adolescents** | | **Adults** |
| **Sample size** | TP1 (baseline; 1 week before) | 281 | 373 | 154 | | 281 |
|  | TP2 (3-24 hrs before) | 184 | 286 | 99 | | 207 |
|  | TP3 (1 day after) | 125 | 226 | 87 | | 188 |
|  | TP4 (2 weeks after) | 90 | 189 | 72 | | 166 |
|  | TP5 (4 weeks after) | 60 | 125 | 52 | | 149 |
| **Age** | | 20.3 ± 2.2 | 35.36 ± 9.52 | 20.6 ± 2.1 | | 36.52 ± 9.3 |
| **Sex** | Male | 224 (79.7%) | 261 (70.0%) | 107 (69.5%) | | 146 (65.5%) |
|  | Female | 55 (19.6%) | 110 (29.5%) | 45 (29.2%) | | 72 (32.3%) |
|  | Other | 2 (0.7%) | 2 (0.5%) | 2 (1.3%) | | 5 (2.2%) |
| **Educational level** | Left school before age 16 without qualifications | 2 (0.71%) | 6 (1.6%) | 2 (1.3%) | | 10 (3.6%) |
|  | High school/GCSE level (UK) | 33 (11.7%) | 12 (3.2%) | 14 (9.1%) | | 9 (3.2%) |
|  | High school diploma/A-Level (UK) | 70 (24.9%) | 27 (7.2%) | 44 (28.6%) | | 23 (8.2%) |
|  | Some university (or equivalent) | 115 (40.9%) | 64 (17.2%) | 52 (33.8%) | | 52 (18.5%) |
|  | Bachelor's degree (or equivalent) | 54 (19.2%) | 139 (37.3%) | 38 (24.7%) | | 91 (32.4%) |
|  | Post-graduate degree (e.g., Masters or Doctorate) | 7 (2.5%) | 125 (33.5%) | 4 (2.6%) | | 96 (34.2%) |
| **Nationality** | United States | 111 (39.5%) | 88 (23.6%) | 44 (28.6%) | | 63 (22.4%) |
|  | United Kingdom | 51 (18.1%) | 77 (20.6%) | 26 (16.9%) | | 89 (31.7%) |
|  | Germany | 18 (6.4%) | 14 (3.8%) | 13 (8.4%) | | 9 (3.2%) |
|  | Canada | 13 (4.6%) | 19 (5.1%) | 10 (6.5%) | | 21 (7.5%) |
|  | Denmark | 10 (3.6%) | 50 (13.4%) | 2 (1.3%) | | 2 (0.7%) |
|  | Mexico | 5 (1.8%) | 1 (0.3%) | 5 (3.2%) | | 3 (1.1%) |
|  | Other (34 in total) | 73 (26.8%) | 124 (33.2%) | 54 (34.1%) | | 94 (33.4%) |
| **Psychiatric history** | Ever been diagnosed with at least one psychiatric illness in the past **^a^** | 89 (31.7%) | 125 (33.5%) | 60 (39.0%) | | 128 (45.6%) |
|  | Never been diagnosed with a psychiatric illness  **^a^** | 192 (68.3%) | 248 (66.5%) | 94 (61.0%) | | 153 (54.4%) |
| **Other previous drug use** | Other drugs - used at least once before **^c^** | 272 (96.8%) | 347 (93.0%) | 149 (96.8%) | | 264 (94.0%) |
|  | Other drugs - never used **^c^** | 9 (3.2%) | 26 (7.0%) | 5 (3.2%) | | 17 (6.0%) |
|  | Alcohol - consumer | 194 (69.0%) | 253 (67.8%) | 109 (70.8%) | | 188 (66.9%) |
|  | Alcohol - non-consumer | 87 (31.0%) | 120 (32.2%) | 45 (29.2%) | | 93 (33.1%) |
| **Previous psychedelic drug use  ^b^** | Never (psychedelic-naïve) | 31 (11.0%) | 31 (8.3%) | 13 (8.4%) | | 37 (13.2%) |
|  | Once | 22 (7.9%) | 18 (4.8%) | 12 (7.8%) | | 16 (5.7%) |
|  | 2-5 times | 87 (31.0%) | 61 (16.4%) | 51 (33.1%) | | 44 (15.7%) |
|  | 6-10 times | 55 (34.1%) | 51 (13.7%) | 33 (21.43%) | | 47 (16.7%) |
|  | 11-20 times | 41 (16.0%) | 68 (18.2%) | 24 (15.6%) | | 47 (16.7%) |
|  | 21 - 50 times | 36 (12.8%) | 74 (19.8%) | 15 (9.7%) | | 47 (16.7%) |
|  | More than 50 times^d^ | 9 (3.20%) | 70 (18.8%) | 6 (3.90%) | | 43 (15.3%) |
| **Bias item “I am a highly experienced psychedelic drug user”** | Strongly disagree | 36 (12.8%) | 45 (12.1%) | 18 (11.7%) | | 50 (17.8%) |
|  | Disagree | 75 (26.7%) | 81 (21.7%) | 51 (33.1%) | | 56 (19.9%) |
|  | Neither agree nor disagree | 78 (27.8%) | 86 (23.1%) | 45 (29.2%) | | 69 (24.6%) |
|  | Agree | 68 (24.2%) | 109 (29.2%) | 28 (18.2%) | | 73 (26.0%) |
|  | Strongly agree | 24 (8.5%) | 52 (13.9%) | 12 (7.8%) | | 33 (11.7%) |

Absolute frequencies with corresponding percentages as well as means ± standard deviations are presented in the table. **^a^** Including major depressive disorder, bipolar disorder, anxiety disorder, schizophrenia, substance abuse disorder, alcohol dependence, hallucinogen persisting perception disorder, psychotic disorder, personality disorder, attention deficit hyperactivity disorder, obsessive compulsive disorder and/or eating disorder. **^b^** Including LSD, DMT/ayahuasca, psilocybin/magic mushrooms/truffles, mescaline (Peyote, San Pedro), Salvia Divinorum, Iboga / Ibogaine, and/or hallucinogen-type NPS. **^c^** Including cannabis, amphetamine, MDMA/ecstasy, cocaine, opiates, benzodiazepines, and/or ketamine. **^d^** Responses “51-100 times” and “More than 100 times” were merged to form “More than 50 times” due to small frequency.

**Supplementary Table 3.** Correlation strength of partial correlations between baseline well-being and previous psychedelic use.

| Measure | Previous psychedelic use | Previous psychedelic use, controlling for other drug use and age |
| --- | --- | --- |
| WEMWBS | **.13**** | **.12*** |
| QIDS | -.05 | -.04 |
| TIPI-ES | -.09 | -.06 |
| BRS | .09 | .09 |
| SCS | **.10*** | **.09*** |
| SIDAS | **-.10*** | **-.12*** |
| STAI-T | **-.11*** | **-.11*** |
| RSE | .08 | .07 |
| CAMS-R | **.18***** | **.17*** |
| SCBCS | .12* | .12* |
| BEA-Q | -.03 | -.01 |
| WCS | **.20*** | **.21*** |

*p<.05, **p<.01, ***p<.001. Analysed using R (*ppcor*::pcor.test, Spearman). *Abbr.*: WEMWBS, Warwick-Edinburg Mental Wellbeing Scale; QIDS, Quick Inventory of Depression Symptoms; TIPI-ES, Ten-Item Personality Inventory-Emotional Stability; BRS, Brief Resilience Scale; SCS, Social Connectedness Scale; SIDAS, Suicidal Ideation Attributes Scale; STAI-T, Spielberger State-Trait Anxiety Inventory; RSE, Roseberg Self-Esteem Scale; CAMS-R, Cognitive and Affective Mindfulness Scale; SCBCS, Santa Clara Brief Compassion Scale; BEA-Q, Brief Experiential Avoidance Questionnaire; WCS, Watt’s Connectedness Scale.

**Supplementary Table 4**. Regression model for ego-dissolution and challenging experiences.

|  | Excluding Retreat | | Excluding Dose | | |
| --- | --- | --- | --- | --- | --- |
|  | *β-*Estimate | *p*-Value | *β-*Estimate | | *p*-Value |
| Ego Dissolution (EDI) | | | | | |
| Fixed Effects: | | | | | |
| Intercept | 18.879 | <.0001*** | 32.282 | | <.0001*** |
| Age grp (Adol.) | 2.325 | .330 | 4.562 | | .066 |
| Dose | 6.637 | <.0001*** | - | | - |
| Retreat (Yes) | - | - | 7.516 | | .005* |
| Bias item | 3.601 | .005** | 4.1.40 | | .002** |
| Previous psychedelic use | -1.721 | -2.056 | -1.364 | | .116 |
| Adj. R^2^: | 0.0734 | | 0.023 | | |
| F-statistic: | F(4, 571)=12.39, p<.0001 | | F(4, 571)=4.305, p=.002 | | |
| Challenging Experiences (CEQ) | | | | | |
| Fixed Effects: | | | | | |
| Intercept | 17.410 | <.0001*** | 22.532 | | <.0001*** |
| Age grp (Adol.) | 2.648 | .060 | 4.477 | | .002** |
| Dose | 3.288 | <.0001*** | - | | - |
| Retreat (Yes) | - | - | 9.980 | | <.0001*** |
| Bias item | -2.120 | .005** | -1.857 | | .0134 |
| Previous psychedelic use | -0.404 | .413 | 0.498 | | <.0001*** |
| Adj. R^2^: | 0.1067 | | | 0.1064 | |
| F-statistic: | F(2, 571)=18.17, p<.0001 | | | F(2, 571)=18.12, p<.0001 | |

*p<.05, **p<.01, ***p<.001, ^†^p<.01. Dose: LSD-equivalent drug dose; Retreat (Yes): being in a psychedelic drug retreat; Bias item: “I am a highly experienced psychedelic drug user”; STAI-T: baseline trait anxiety. **Bold**: age group significantly predicts EDI after removing dose from the model.

**Supplementary Table 5**. Coefficient estimates and their predictive significance of age group on CEQ subscale scores, and corresponding Pearson correlations.

| Fixed Effect: Age group (Adolescents) | All Predictors | | Pearson correlations | |
| --- | --- | --- | --- | --- |
|  | *β-*Estimate | *p*-Value | r | *p*-Value |
| Fear | 0.264 | .002** | .132 | .002** |
| Physical distress | 0.314 | <.001*** | .131 | .002** |
| Insanity | 0.239 | .005** | .128 | .002** |
| Isolation | 0.200 | .022* | .111 | .008** |
| Paranoia | 0.236 | .010* | .128 | .002** |

*p<.05, **p<.01, ***p<.001. *Abbr.*: CEQ, Challenging Experiences Questionnaire.
